# No preliminary evidence of differences in astrocyte density within the white matter of the dorsolateral prefrontal cortex in autism

**DOI:** 10.1186/s13229-017-0181-5

**Published:** 2017-12-08

**Authors:** Ting Ting Lee, Efstratios Skafidas, Mirella Dottori, Daniela Zantomio, Christos Pantelis, Ian Everall, Gursharan Chana

**Affiliations:** 1 Department of Psychiatry, The University of Melbourne, Parkville, Australia; 2 Centre for Neural Engineering, The University of Melbourne, Parkville, Australia; 3 Department of Biomedical Engineering, The University of Melbourne, Parkville, Australia; 4 Department of Clinical Haematology, Austin Hospital, Heidelberg, Australia; 5 Department of Medicine, The University of Melbourne, Parkville, Australia; 6 Melbourne Neuropsychiatry Centre, The University of Melbourne & Melbourne Health, Parkville, Australia; 7 Florey Institute of Neuroscience and Mental Health, The University of Melbourne, Parkville, Australia; 8 The Institute of Psychiatry, Psychology and Neuroscience, King’s College London, London, UK; 9 Illawarra Health and Medical Research Institute, University of Wollongong, Wollongong, Australia

**Keywords:** Astrocytes, Glia, Autism, Dorsolateral prefrontal cortex (DLPFC), Cell density, White matter

## Abstract

**Background:**

While evidence for white matter and astrocytic abnormalities exist in autism, a detailed investigation of astrocytes has not been conducted. Such an investigation is further warranted by an increasing role for neuroinflammation in autism pathogenesis, with astrocytes being key players in this process. We present the first study of astrocyte density and morphology within the white matter of the dorsolateral prefrontal cortex (DLPFC) in individuals with autism.

**Methods:**

DLPFC formalin-fixed sections containing white matter from individuals with autism (*n* = 8, age = 4–51 years) and age-matched controls (*n* = 7, age = 4–46 years) were immunostained for glial fibrillary acidic protein (GFAP). Density of astrocytes and other glia were estimated via the optical fractionator, astrocyte somal size estimated via the nucleator, and astrocyte process length via the spaceballs probe.

**Results:**

We found no evidence for alteration in astrocyte density within DLPFC white matter of individuals with autism versus controls, together with no differences in astrocyte somal size and process length.

**Conclusion:**

Our results suggest that astrocyte abnormalities within the white matter in the DLPFC in autism may be less pronounced than previously thought. However, astrocytic dysregulation may still exist in autism, even in the absence of gross morphological changes. Our lack of evidence for astrocyte abnormalities could have been confounded to an extent by having a small sample size and wide age range, with pathological features potentially restricted to early stages of autism. Nonetheless, future investigations would benefit from assessing functional markers of astrocytes in light of the underlying pathophysiology of autism.

**Electronic supplementary material:**

The online version of this article (10.1186/s13229-017-0181-5) contains supplementary material, which is available to authorized users.

## Background

Autism is a highly complex and heritable neurodevelopmental disorder with prevalence rates soaring from 1 in 5000 children in the 1970s [1] to 1 in 132 global prevalence reported in recent years [2]. The majority of post-mortem brain studies in autism have focused on uncovering neuronal abnormalities; however, recent evidence has demonstrated alterations in both microglial and astrocytic markers within the autistic brain [3]. Microglia and astrocytes are known to have crucial roles in neurodevelopment, including regulation of synapse formation as well as neuroinflammatory and immune pathways. As both synaptogenesis disruption and neuroinflammation are reported in autism, a role for microglial and astrocyte involvement in the pathophysiology of autism has begun to receive an increasing amount of attention [3–5].

Astrocytes represent the most abundant glia cell type in the brain, and in their mature state, form tripartite synapses together with presynaptic and postsynaptic neurons in a three-way interaction to regulate neuronal signaling via neurotransmitter/gliotransmitter receptors and ion channels at the level of a single synapse or a neuronal network [3, 6–8]. Astrocytes have also been shown to play a critical role in oligodendrocyte survival and maturation, including influencing the ability of oligodendrocytes to myelinate axons [9, 10]. In addition, glial fibrillary acidic protein (GFAP), a major structural component of astrocytes, has been shown to be necessary for the integrity of CNS white matter as well as long-term maintenance of myelination [11]. This is of interest given post-mortem studies have revealed axonal thinning within the white matter in individuals with autism as well as reduced myelin thickness in the anterior cingulate cortex (ACC) and prefrontal cortex (PFC) [12]. While neuroimaging studies have shown accelerated maturation of white matter within the frontal cortex [13, 14], increased gray and white matter volume in the frontal and temporal regions [15–18], as well as hyper-connectivity in prefrontal, amygdala, and temporal regions [18, 19]. Along with the ACC, the dorsolateral prefrontal cortex (DLPFC) is responsible for higher level executive functioning, including attention, emotional processing, learning and memory, domains that are impaired in individuals with autism [20]. This is evidenced by several studies reporting impairments in individuals with autism compared to controls when performing tasks known to be mediated by the DLPFC, including poor performance in the Wisconsin Card Sorting Test (WCST), as well as decreased functional activity as measured by magnetic resonance spectroscopy (MRS) and functional magnetic resonance imaging (fMRI) [21–23].

Evidence for astrocyte dysfunction have also come from a post-mortem brain investigation in autism demonstrating increased mRNA levels of *GFAP* as well as the excitatory amino acid transporter 1 (*EAAT1*) via microarray profiling within the cerebellum, with upregulation confirmed at the protein level via western blotting [24]. Subsequently, Vargas et al. demonstrated increased GFAP in the cerebellum, middle frontal gyrus, and ACC within individuals with autism compared to controls [25]. This was followed by Laurence and Fatemi’s results, also demonstrating increased protein levels of GFAP in the superior frontal, parietal and cerebellar cortex in autism [26]. Further support for prefrontal and cerebellar changes were demonstrated by a quantitative real-time polymerase chain reaction (qPCR) investigation showing increased *GFAP* expression in both regions in autism compared to controls [27]. In addition, Crawford et al. demonstrated increased GFAP at the protein level via western blotting within the white matter of the anterior cingulate gyrus, but not in the gray matter [28]. In contrast to increased GFAP levels within the brain in autism, Morgan et al. demonstrated no difference in density and size of astrocytes between autism and controls following a stereological study of the amygdala [29]**.** Other astrocytic abnormalities have also been observed in the brains of individuals with autism, including reduced protein levels of aquaporin 4 (AQP4) within the cerebellum together with elevated connexin 43 (CNX43) seen in the superior frontal cortex [30].

Astrocytes are known to be intimately involved in the inflammatory response within the CNS [31], with growing evidence implicating neuroinflammation in the pathophysiology of autism, especially via dysregulation of neurogenesis and neuronal physiology related to activation of microglia and astrocytes [5, 32, 33]. Astrocyte activation in autism has been suggested to occur due to observations of astrogliosis in the cerebellar white matter and subcortical white matter within the frontal lobe [25–27]. Given that astrocytes are known to be involved in neurodevelopment, potential changes in their activation state and/or functioning due to inflammation during development might have consequences for disrupted connectivity and neuropathological changes in autism [34]. Activation of astrocytes and the subsequent pro-inflammatory cytokines release, including interleukin 6 (IL-6), tumor necrosis factor alpha (TNFα), and interleukin 1B (IL-1B), has also been observed in autism within the brain as well as peripherally in the cerebrospinal fluid [25, 35].

Given the growing body of evidence for astrocytic involvement in autism, and their known functioning in maintaining myelin, together with disruptions in myelin integrity seen within the brain of individuals with autism, a more detailed investigation of astrocytic neuropathology within the white matter is warranted. The aim of this investigation was to assess astrocyte density and morphology within the white matter of the DLPFC in autism compared to age- and gender-matched controls using GFAP as a cellular astrocyte marker. This was achieved using the optical fractionator, nucleator, and spaceballs probes to investigate density, somal size, and overall process length respectively.

## Methods

### Brain tissue

Formalin-fixed DLPFC tissue blocks restricted to BA9 and BA46 from individuals with autism (*n* = 11) and controls (*n* = 10) were provided by the NICHD Brain and Tissue Bank for Developmental Disorders (NBTBD), Baltimore, Maryland upon request (University of Melbourne ethics approval ID: 1339835.1). Tissue was matched as closely as possible for age between autism (range = 4–51 years) and controls (range = 4–46 years). Tissue blocks were serially sectioned using a Leica VT1000s Vibratome (Leica Biosystems, Germany) in phosphate-buffered saline (PBS) at 50 μM and stored in PBS with 0.5% sodium azide at 4 °C. Six samples (3 autism, 3 controls) were excluded from analysis due to poor tissue morphology following immunohistochemistry, thereby reducing the number of cases available to 8 autism and 7 controls (Table 1).

**Table 1:** Brain tissue demographics

Sample ID	Age		Cause of death	Sex	PMI (h)	Diagnosis
	Years	Days				
5308	4	182	Skull fractures	M	21	ASD
5144	7	55	Complications from cancer	M	3	ASD
4899	14	126	Drowning	M	9	ASD
5403	16	266	Cardiac arrhythmia	M	35	ASD
4999	20	274	Cardiac arrhythmia	M	14	ASD
5578	35	294	Accident	M	22	ASD
5115	46	135	Complications of pseudomyxoma peritonei	M	29	ASD
5137	51	91	Pneumonia	M	72	ASD
1185	4	258	Drowning	M	17	Control
1500	6	320	Multiple injuries	M	18	Control
917	14	217	Accident/multiple injuries	M	10	Control
1158	16	63	Cardiomegaly	M	15	Control
4916	19	47	Accident/drowning	M	5	Control
4593	33	24	Cardiac arrhythmia	M	8	Control
1936	46	35	Arteriosclerotic cardiovascular disease	M	13	Control

### Immunohistochemistry

Four sections from each subject were chosen via systematic random sampling (every 14 sections) spanning the entire tissue blocks and processed for immunohistochemistry using a Vectastain ABC Kit (Vector Laboratories, USA) and a rabbit polyclonal antibody against GFAP (DAKO, Denmark) [1:4000]. For detailed methods, please refer to Additional file 1.

### Astrocyte density and morphological measurement

All density quantitation was performed using a ×100 oil immersion objective lens (numerical aperture = 1.4) and a Nikon Eclipse 80i (Nikon Instruments, Japan) together with StereoInvestigator 11 (MBF Biosciences, USA) as described previously [33]. A pilot study was conducted to determine sampling parameters of probes including disector depth, size of sampling contour, and numbers of sampling fields in order to achieve coefficient of error (CE) below 0.05 [36]. Three regions of interest defined by 1 mm × 2 mm (optical fractionator and nucleator) or 1 mm × 1 mm (spaceballs) were drawn using ×4 objective lens within the white matter for each section and systematic random sampling (SRS) grid applied for data collection. Within StereoInvestigator, we utilized the optical fractionator probe to estimate astrocyte and glia density, with simultaneous application of the nucleator probe for astrocyte somal size estimation. The spaceballs probe was used to estimate total astrocyte process length. For detailed methods, please refer to Additional file 1.

### Statistical analysis

All measures were tested for normality using a Shapiro-Wilk test, with Pearson or Spearman correlations, carried out between our outcome measures, age, and post-mortem interval (PMI). Any significant correlation at *p* < 0.05 were considered as covariates during analysis for group differences. A general linear model was used to test differences between groups for normally distributed measures while a Mann-Whitney *U* test was utilized for non-parametrically distributed measures.

## Results

### Identification of adjustment variables

Shapiro-Wilk normality tests demonstrated that nucleator area (*p* = 0.023) was not normally distributed within the autism group; however, all other measures including age, post-mortem interval (PMI), astrocytes density, negative glia density, total glia density, spaceballs length, and IOD were normally distributed. No significant correlations were observed (Table 2).

**Table 2:** Statistical analysis of normality test, correlation test, and group comparison between autism and control

	Diagnosis	Shapiro-Wilk normality test			Correlation age		Correlation PMI		Group differences (ASD vs control)		
		Statistic	df	Sig.	Pearson (*r*)	Spearman (rho)	Pearson (*r*)	Spearman (rho)	Mean	SE	*p* value
Age	ASD	0.907	8	0.335					24.612	5.803	0.604^a^
	Control	0.907	7	0.374					20.092	6.204	
PMI (h)	ASD	0.864	8	0.133					25.625	5.673	0.132^a^
	Control	0.954	7	0.766					12.286	6.065	
Population (all glia)	ASD	0.950	8	0.715	0.205 (*p* = 0.463)		− 0.390 (*p* = 0.150)		2,401,704.83	121,494.58	0.415^a^
	Control	0.873	7	0.196					2,351,803.21	129,883.17	
Population (astrocyte)	ASD	0.969	8	0.894	− 0.267 (*p* = 0.337)		0.168 (*p* = 0.549)		100,669.51	8557.62	0.202^a^
	Control	0.978	7	0.949					117,513.34	9148.48	
Population (other glia)	ASD	0.951	8	0.718	0.219 (*p* = 0.433)		− 0.392 (*p* = 0.149)		2,401,035.25	124,249.39	0.376^a^
	Control	0.868	7	0.178					2,234,289.88	132,828.19	
Nucleator (area)	ASD	0.791	8	0.023		− 0.093 (*p* = 0.742)		− 0.118 (*p* = 0.676)	92.475	4.556	0.397^b^
	Control	0.866	7	0.170					87.423	4.870	
Spaceballs length (all) (um)	ASD	0.901	8	0.298	− 0.289 (*p* = 0.296)		− 0.206 (*p* = 0.461)		51,228,155.8	6,563,846.09	0.923^a^
	Control	0.982	7	0.970					52,176,610.3	7,017,046.63	
IOD (mean)	ASD	0.938	8	0.591	− 0.681 (*p* = 0.007)		− 0.517 (*p* = 0.059)		143.083	4.159	0.066^c^
	Control	0.922	7	0.487					156.127	4.809	

^a^General linear model

^b^Non-parametric independent sample *t* test

^c^General linear model with covariate “age”

### Astrocyte and total glial densities

**Fig. 1:**
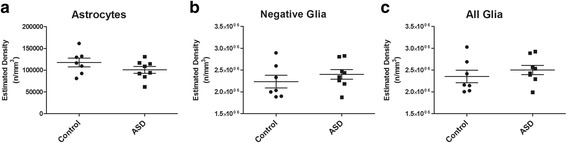
Optical fractionator results of ASD and controls. Estimated density of **a** astrocytes (GFAP positive), **b** negative glia (GFAP-negative glia), and **c** total glia, in the DLPFC of ASD and control subjects

No significant differences in the density of astrocytes were seen between autism and controls (autism = 10.07 × 10^4^ ± 85.58 × 10^2^, control = 11.75 × 10^4^ ± 91.48 × 10^2^ (mean ± SEM); *p* = 0.202, Fig. 1a). In addition, no group differences were seen for GFAP negative glia (autism = 24.01 × 10^5^ ± 12.42 × 10^4^, control = 23.34 × 10^5^ ± 13.28 × 10^4^ (mean ± SEM); *p* = 0.376, Fig. 1b), or total glia (autism = 24.02 × 10^4^ ± 12.15 × 10^4^, control = 23.52 × 10^4^ ± 12.99 × 10^4^ (mean ± SEM); *p* = 0.415, Fig. 1c).

### Astrocyte somal size and process length

**Fig. 2:**
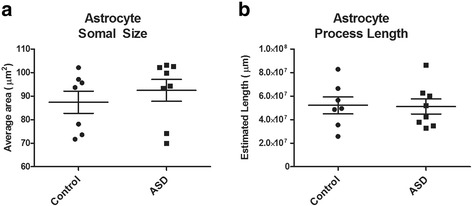
Astrocyte morphology estimates. Astrocytes reactivity measures of **a** cell somal area via nucleator probe, and **b** cell processes length using spaceballs probe

As somal sizes were not normally distributed within the autism group, we employed a Mann-Whitney *U* non-parametric test to compare differences between autism and control groups. No significant difference between autism and controls were seen for somal area of white matter astrocytes (autism = 92.475 ± 4.56 μm^2^, control = 87.423 ± 4.87 μm^2^ (mean ± SEM); *p* = 0.397, Fig. 2a). Furthermore, spaceballs analysis showed no significant difference between autism and control group for total length of astrocytes processes in the white matter (autism = 51.23 × 10^6^ ± 65.64 × 10^5^ μm, control = 52.18 × 10^6^ ± 70.17 × 10^5^ μm (mean ± SEM); *p* = 0.923, Fig. 2c).

## Discussion

This study provides the first investigation of astrocyte density, morphology, and activation status within the white matter of the DLPFC in autism. Based on previous evidence demonstrating increased GFAP within the brain in autism, we hypothesized that we would observe an increased density of astrocytes in the white matter of the DLPFC of individuals with autism. In contrast, our results demonstrate no difference in the density and cellular morphology of GFAP-positive astrocytes in the white matter of the DLPFC of autism when compared to age-matched controls. Our findings are in keeping with recent studies demonstrating no alteration in astrocyte number and astrocyte somal size in the amygdala in autism, as well as no increase in GFAP mRNA and protein in the anterior PFC and ACC [28, 29].

During severe reactive astrogliosis, a marked increase in astrocytic size, arborization, GFAP expression, and proliferation are observed [37, 38]. Therefore, the current study investigated the population of GFAP-positive astrocytes along with other glial cells using StereoInvestigator to obtain an estimation of astrocyte and glial density, as well as astrocytic somal size and process length. Our results provide support for no alteration in astrocyte or glial density in the autism brain, consistent with Morgan et al. reporting no alteration of astrocyte numbers in autism within the amygdala [29]. In addition, our results demonstrated no evidence for increased astrocytic reactivity within the DLPFC white matter of individuals with autism compared to controls, as indicated by no changes in morphology, including cell somal size and process length. Similar to our findings for astrocyte somal size, Morgan et al. also found no evidence for differences in astrocyte somal size between autism and controls within the amygdala [29].

Our lack of findings for alterations in astrocyte density and activation status within the DLPFC white matter in autism represent an absence in gross astrocyte morphological changes in this brain region. However, another possibility is that any astrocyte activation may be present at a less pronounced level and that our employed strategies for investigating activation may have not been sensitive enough to detect such changes. This is in keeping with a lack of stark neuropathological changes observed in the autism brain. Yet another possible explanation is that astrocytes measured for our investigation may have resolved their reactivity and activation status, with morphological changes potentially taking place much earlier in disease progression [38]. Given the early developmental nature of autism and likely heterogeneity in autism pathophysiology, the latter two possibilities are equally likely to potentially explain the lack of astrocyte reactivity within our samples. Our results therefore highlight the need for more sensitive measures of assessing astrocyte activation as well as potentially assessing temporal and regional changes in astrocyte activation.

Despite a lack of evidence in our study to suggest activation of astrocytes, their contribution to the pathophysiology of autism might still be very significant. Given astrocytes play a central role in regulating the functions of other cell types in the brain, any loss or gain of function of astrocytes could have a direct or indirect effect on brain physiology. Astrocytes contribute to synaptic functioning, especially clearing of glutamate via transporter EAAT2. A disturbance of this function could potentially be detrimental to neuronal health and functioning due to excitotoxicity caused by excess glutamate within the extracellular space [39]. Developmentally, astrocytes contribute to synaptic pruning by actively removing synapses via MEFG10 and MERTK pathways [40], or by tagging synapses for elimination by microglia via upregulation of C1q protein, loss of this function could lead to over- or under-pruning of synapses as observed in autism [41]. Within the white matter, disturbance of astrocytic physiology could also lead to perturbation of myelination, which has also been observed in autism [42, 43]. Therefore, potential functional alterations in astrocytes due to neuroinflammation and change in their activation may warrant a more detailed investigation in an attempt to tease out the mechanistic roles of astrocytes in the neuropathology of autism.

Due to the limitation of post-mortem brain samples available, autism studies including the current one are often limited by sample size. This also contributed to a relatively large age range within the cohort investigated. Consequently, our lack of astrocyte abnormalities in the current study could to a certain extent have been masked by an age-related effect, whereby abnormalities present in early developmental stages may be normalized later in life. This is particularly interesting given age-related cortical thinning has been reported within the temporal and parietal cortices of autism brain during adolescence and early adulthood, despite evidence of cortical overgrowth earlier in life [44]. In addition, findings from several longitudinal neuroimaging studies also suggest that brain abnormalities such as overgrowth is pronounced within the first few years of life and subsequently found to normalize towards controls during aging [45–47]. Furthermore, it is not uncommon for individuals with autism to have comorbid neurological disorders such as epilepsy, which may additionally represent a confounding factor, with potential brain changes due to an underlying neurodegenerative disease. With regard to epilepsy, this would be particularly pertinent for our investigation as individuals with epilepsy are known to have increased astrocyte numbers and activation [48].

For this study, we employed a strict protocol of sampling whereby we ensured the CE for all cases was below 0.05, in attempt to increase our precision in estimating density, size, and process length measurements. As the spaceballs probe was used to estimate all astrocyte processes present within the ROI regardless of the presence of the cell body, our results reflect more global increases in astrocyte length as opposed to arborization characteristics of individual astrocytes. Nevertheless, our data provides a reliable 3D estimate of overall length of astrocyte processes as a measure of potential astrocyte activation. Future studies may benefit from investigating an extended suite of astrocyte markers, including aldehyde dehydrogenase 1 family member L1 (ALDH1L1), glutamate synthetase (GS), and S100 calcium-binding protein B (S100B), to obtain a more complete perspective on structural and functional astrocyte abnormalities in the autism brain.

## Conclusions

In conclusion, our results suggest that gross morphological changes in astrocyte density and activation status are not present in the DLPFC white matter. This could suggest a lack of astrogliosis, but does not rule out astrocytic abnormalities, that may be present in the absence of gross morphological changes. In addition, regional differences in the reactivity of astrocytes in the brain in autism may also be present and should be considered. Further post-mortem investigations within the autism brain should be aimed at assessing astrocyte abnormalities utilizing more functional markers of astrocytes to further understand the role that astrocytes play in the pathophysiology of autism and their impact on other cell types in the brain including neurons, microglia, and oligodendrocytes, with these cells also likely contributing to the underlying etiology of the disorder.

## Additional file

Additional file 1: Supplemental methods. (DOCX 12083 kb)

## Abbreviations

ABC: Avidin-biotin complex
ACC: Anterior cingulate cortex
ALDH1L1: Aldehyde dehydrogenase 1 family member L1
AOI: Area of interest
AQP4: Aquaporin 4
CE: Coefficient of error
CNS: Central nervous system
CNX43: Connexin 43
DAB: Diaminobenzadine
DLPFC: Dorsolateral prefrontal cortex
EAAT1: Excitatory amino acid transporter 1
GFAP: Glial fibrillary acidic protein
GS: Glutamate synthetase
IL-1B: Interleukin 1B
IL-6: Interleukin 6
IOD: Integrated optical density
MRI: Magnetic resonance imaging
NGS: Normal goat serum
PBS: Phosphate-buffered saline
PFC: Prefrontal cortex
qPCR: Quantitative real-time polymerase chain reaction
S100B: S100 calcium binding protein B
SEM: Standard error of mean
SRS: Systematic random sampling
TNFα: Tumor necrosis factor alpha
