# Supplementary material for: No preliminary evidence of differences in astrocyte density within the white matter of the dorsolateral prefrontal cortex in autism
Source: Mol Autism. 2017 Dec 8;8:64. doi: 10.1186/s13229-017-0181-5 (PMC5721546; doi:10.1186/s13229-017-0181-5)
Supplement: Additional file 1: — Supplemental methods. (DOCX 12083 kb) [file 13229_2017_181_MOESM1_ESM.docx]

No Preliminary Evidence of Differences in Astrocyte Density within the White Matter of the Dorsolateral Prefrontal Cortex in Autism

Authors:

Ting Ting Lee ^1,2^, Efstratios Skafidas ^1,2,6^, Mirella Dottori ^2,3^, Daniela Zantomio^4,^ Christos Pantelis ^1,5,6^, Ian Everall* ^1,2,6^, Gursharan Chana* ^1,2,4,6^

1 Department of Psychiatry, The University of Melbourne, Australia.

2 Centre for Neural Engineering, The University of Melbourne, Australia.

3 Department of Biomedical Engineering, The University of Melbourne, Australia.

4 Department of Haematology, Austin Health, Heidelberg, Vic, Australia

5 Department of Medicine, The University of Melbourne, Australia.

6 Melbourne Neuropsychiatry Centre, The University of Melbourne, Australia.

7 Florey Institute of Neuroscience and Mental Health, University of Melbourne Parkville, Victoria, Australia

* Authors contributed equally

Corresponding author:

Ting Ting Lee

203 Bouverie Street, Carlton VIC 3053 Australia

[tingtl@unimelb.edu.au](mailto:tingtl@unimelb.edu.au)

(61) 03 9035 9818

**Supplemental Methods**

Immunohistochemistry

Free-floating sections were incubated in 2% hydrogen peroxide (Chem-Supply, Australia) in 100% methanol (Chem-Supply, Australia) for 20 minutes at room temperature. Following removal of excess methanol and hydrogen peroxide through washing in phosphate buffered saline (PBS), sections were microwaved in 1X citrate buffer [0.01% hydrochloric acid] (Sapphire Biosciences, Australia) at 1000W for 3 minutes, then at 500W for another 3 minutes in order to aid antigen retrieval. Sections were then incubated in blocking solution consisting 3% normal goat serum (NGS) in PBS containing 0.1% Triton-X100 (Sigma, USA) for 1 hour at room temperature, followed by incubation with a rabbit polyclonal antibody against GFAP (DAKO, Denmark) [1:4000] in 1.5% normal NGS in PBS containing 0.1% Triton-X100 overnight at 4⁰C. Sections were washed three times in excess PBS, followed by incubation in a biotinylated secondary anti-rabbit immunoglobulin G (IgG) 1.5% normal NGS in PBS containing 0.1% Triton-X100 for 2 hours at room temperature. Sections were then washed again three times in excess PBS and then incubated in Avidin-Biotin Complex (ABC) for 1 hour at room temperature. Sections were washed again in PBS three times and immunostaining visualized using a ImmPACT diaminobenzadine (DAB) peroxidase substrate (Vector Laboratories, USA). Following washing in PBS and distilled water, sections were counterstained using haematoxylin (Vector Laboratories, USA) and mounted onto Menzel-Glaser Superfrost® Ultra Plus microscope slides (Thermo Scientific, Germany) and allowed to air dry overnight. Sections were then gradually dehydrated in graded ethanol (Chem-Supply, Australia), and then in xylene (Chem-Supply, Australia), followed by permanent mounting using DPX mountant (Sigma Aldrich, USA).

Stereological quantitation

*Regions of interest (ROI)*

Regions of interest (ROI) were chosen within the DLPFC where the pia matter, grey matter, and white matter could be clearly identified. Using Stereo Investigator 11, three contours defining our ROI 1mm x 2mm (x- and y- axes) (optical fractionator & nucleator) or 1mm x 1mm (spaceballs) were drawn using a 4x objective lens within the white matter for each section, using the edge of white/grey matter as a reference point (Supplemental figure 1). Due to the different sizes, regions, and composition on grey/white matter of tissue blocks and sections, the pilot study determined the largest possible ROI that could be achieved across all sections and cases. ROI contour traces were overlaid between subsequent sections and cases together with a constant grid size maintained as a pre-requisite within the optical fractionator to enable computation of Gunderson’s CE. Following delineation of our ROIs, StereoInvestigator 11 then systemically overlaid optical disectors representing our individual fields for sampling.

*Identification of cells*

Astrocytes, negative glia and neurons were identified with criteria as described in previous studies (1). Briefly, astrocytes were identified by positive GFAP staining in the membrane, cytoplasm and nucleus as described in the human protein atlas (2, 3). Negative glia was identified via a rounded nucleus, lack of visible cytoplasm and the presence of heterochromatin. Neurons were identified based on their larger size, counterstained nucleus and visible nucleolus.

*Optical Fractionator*

Our pilot analysis revealed that our processed and mounted sections had an average thickness between 19.5µm to 24.7µm, with the lowest single-point thickness recorded at 16.8µm. An optical disector depth of 12µm was chosen based on our lowest z-axis measure, and to allow for a 2μm upper guard zone, in order to eliminate z-axis bias due to unevenness of the sections. Pilot analysis revealed that approximately 40 sampling fields were required to obtain a CE below 0.05 (4). An optical disector of 70µm x 70µm x 12µm (x,y, and z-axes) was employed (Supplemental figure 2), giving a total disector volume of 58.8 x 10^3^ µm^3^ per sampling field. Disectors where more than 50% of the sampling field did not contain any tissue, due to tear or hole in section, blood vessels (to minimize bias of potentially sampling more or less blood vessels and perivascular astrocytes, due to plane of sectioning), and outside of the AOI contour were excluded to prevent bias in estimation of cell counts. Estimation of densities of GFAP positive astrocytes, GFAP negative glia, and total number of glia were then calculated for our AOI of 1mm x 2mm for each case.

*Nucleator*

Somal size of positively identified cells were measured using the nucleator probe with 6 random isotropic rays spanning the soma radiating from the center and marked manually at the edges. This was performed simultaneously within the optical fractionator workflow, with average somal size estimated for each section and sample. (Supplemental figure 3). Nucleator measurements for all samples had CE’s lower than 0.005.

*Spaceballs*

The spaceballs probe within Stereo Investigator 11 was employed to estimate the length of astrocyte processes for our region of interest. Pilot analysis revealed that approximately 30 systematically random sampled (SRS) fields were required to collect measurements with CE lower than 0.05. Using the spaceballs probe, a virtual hemisphere was embedded within the tissue and each transection of astrocytes processes with the outline of hemisphere was recorded (Supplemental figure 4) and the total astrocyte process lengths within the 30 SRS fields in the region of interest estimated. Sampling field was excluded if more than 50% of virtual hemisphere overlay contained no tissue.

**Supplemental Figures**

**
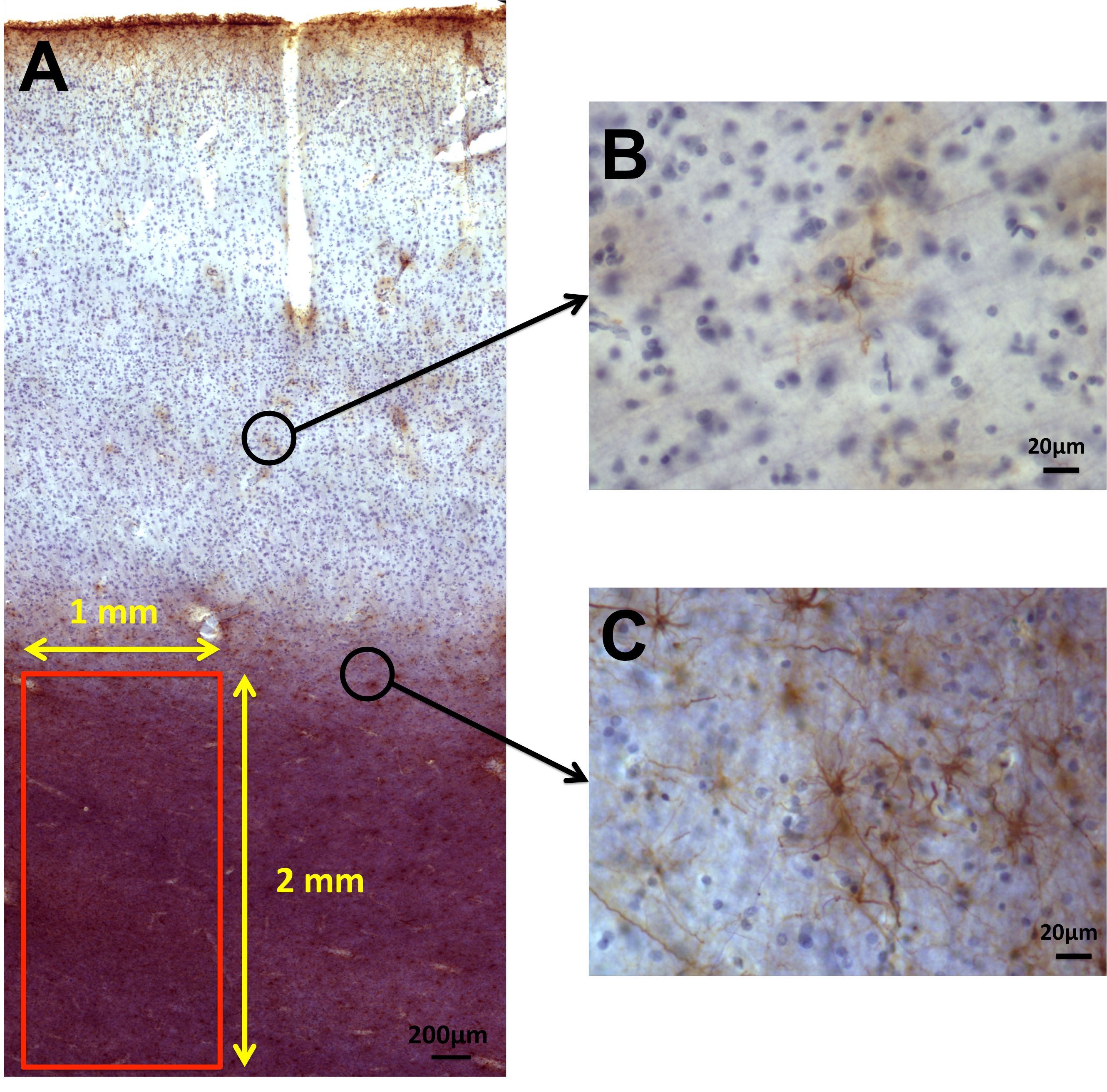
**

**Supplemental figure 1**. **(A)** Tiled images captured at 10x objective, from control sample 4916, showing GFAP staining in radial glial cells near the pia, as well as grey and white matter astrocytes. An area of interest (AOI) was drawn with 1mm width across the grey and white matter border, extending 2mm into the white matter. 40 sampling fields were generated within the AOI using systematic random sampling (SRS) method within Stereo Investigator 11. **(B)** Enlarged grey matter area from tiled image showing GFAP positive astrocyte at 40x objective. **(C)** Enlarged white matter area from tiled image showing GFAP positive astrocytes at 40x objective.


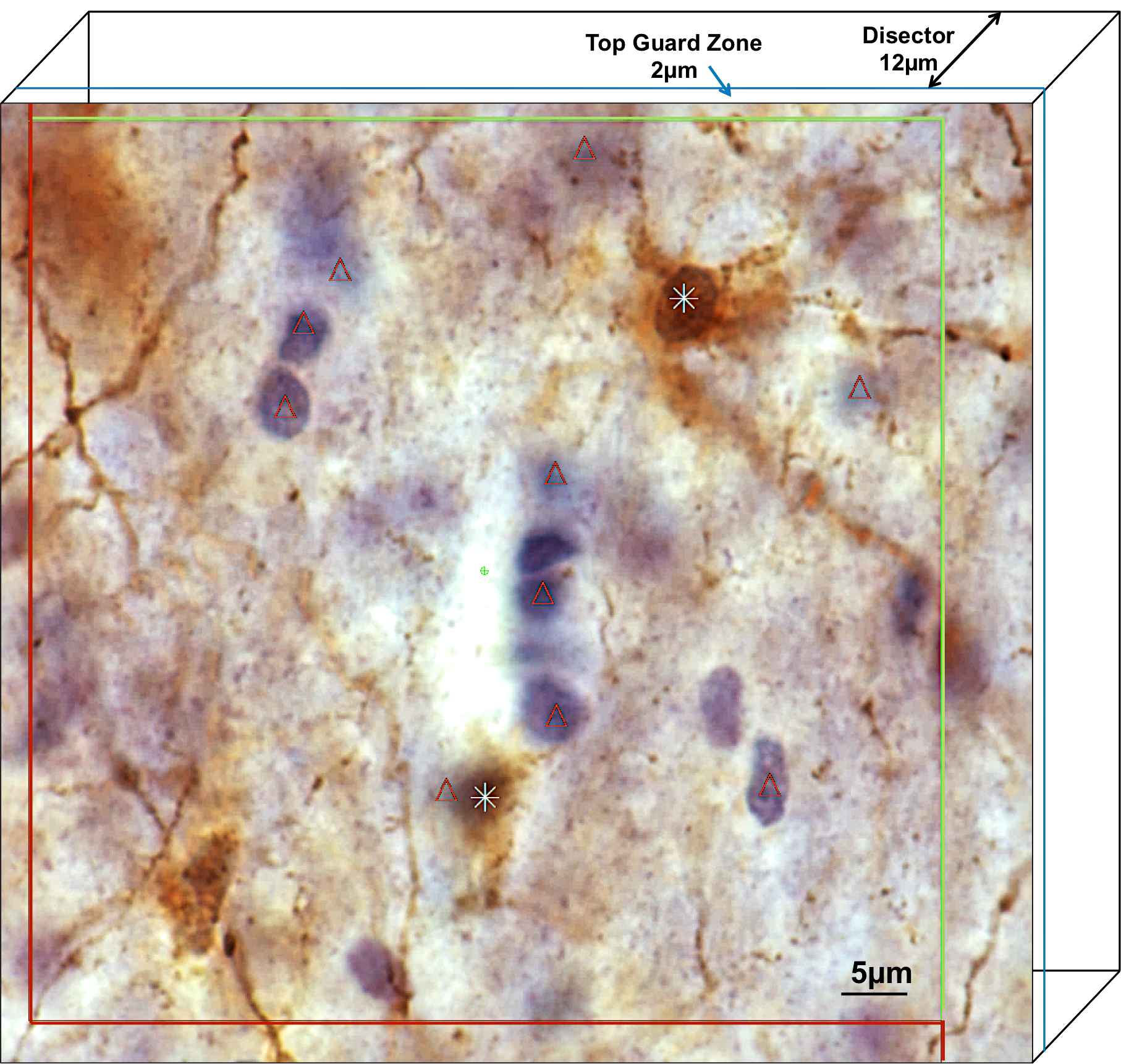


**Supplemental figure 2**. Optical disector represented by a rectangular cuboid of 70µm x 70µm x 12µm (width x height x depth), defined by 2 inclusion lines (green), 2 exclusion lines (red) on the x- and y-axes, center point of disector marked by green dot, as well as disector depth (black line with double arrowhead) of 12µm on the z-axis. Top guard zone (blue arrow and lines) of 2µm was employed to account for bias due to unevenness of tissue. Astrocytes (white asterisk) were identified via a GFAP stained cell body, processes, and nucleus. Any white matter neurons were identified via the presence of a single nucleolus, larger appearance and non-spherical shape as well as counterstain in the cytoplasm. Other glial cells were identified via their smaller nucleus, spherical appearance, and the presence of heterochromatin as well as a lack of visible cytoplasm (red triangle). Cells were only counted if nucleus (glia) or nucleolus (neurons) were in focus within the disector. Images captured using a 100x objective (n.a. = 1.4).


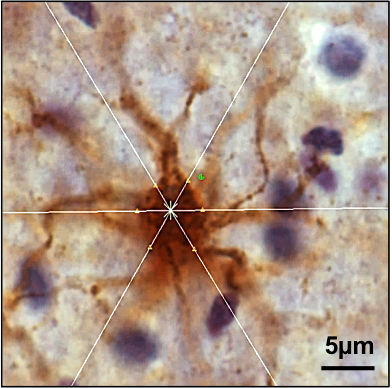


**Supplemental figure 3.** Nucleator probe with 6 isotropic rays radiating from the centre of the cell soma. Investigator tags edges of cells so that software can measure the diameters and hence use the information to estimate somal size. 100x objective (n.a. = 1.4).


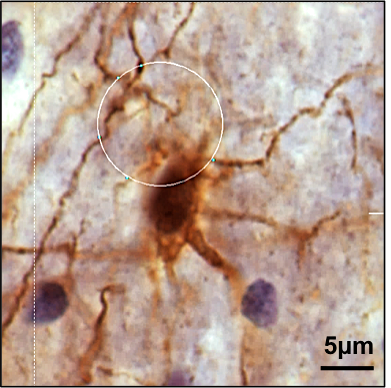


**Supplemental figure 4.** Spaceballs probe using hemisphere, any intersection point between the outline of hemisphere and astrocytes processes in focus were marked, regardless of the presence of cell body. Image captured at 100x objective (n.a. = 1.4).

1. Chana G, Laskaris L, Pantelis C, Gillett P, Testa R, Zantomio D, et al. (2015): Decreased expression of mGluR5 within the dorsolateral prefrontal cortex in autism and increased microglial number in mGluR5 knockout mice: Pathophysiological and neurobehavioral implications. *Brain, behavior, and immunity*. 49:197-205.

2. Uhlén M, Fagerberg L, Hallström BM, Lindskog C, Oksvold P, Mardinoglu A, et al. (2015): Tissue-based map of the human proteome. *Science (New York, NY)*. 347.

3. Pontén F, Jirström K, Uhlen M (2008): The Human Protein Atlas—a tool for pathology. *The Journal of Pathology*. 216:387-393.

4. Gundersen HJ, Jensen EB, Kieu K, Nielsen J (1999): The efficiency of systematic sampling in stereology--reconsidered. *Journal of microscopy*. 193:199-211.
